# Identification of Near Geographical Origin of Wolfberries by a Combination of Hyperspectral Imaging and Multi-Task Residual Fully Convolutional Network

**DOI:** 10.3390/foods11131936

**Published:** 2022-06-29

**Authors:** Jiarui Cui, Kenken Li, Jie Hao, Fujia Dong, Songlei Wang, Argenis Rodas-González, Zhifeng Zhang, Haifeng Li, Kangning Wu

**Affiliations:** 1 School of Food and Wine, Ningxia University, Yinchuan 750021, China; c15145244344@163.com (J.C.); likenk_123@163.com (K.L.); jiehao0518@163.com (J.H.); fujiadongn@yeah.net (F.D.); lihaifeng0426@nxu.edu.cn (H.L.); 2 Department of Animal Science, Faculty of Agricultural and Food Sciences, University of Manitoba, Winnipeg, MB R3T 2N2, Canada; admissions@umanitoba.ca; 3 School of Food Science and Technology, Huazhong Agricultural University, Wuhan 430070, China; xb@mail.hzau.edu.cn; 4 Ningxia Huaxinda Health Science and Technology Co., Ltd., Lingwu 751400, China; kningwu@163.com

**Keywords:** hyperspectrum, wolfberry, origin identification, fusion of spectral and image, Bayesian optimization, denoising auto-encoder, up-sampling, deep learning, multitask

## Abstract

Ningxia wolfberry is the only wolfberry product with medicinal value in China. However, the nutritional elements, active ingredients, and economic value of the wolfberry vary considerably among different origins in Ningxia. It is difficult to determine the origin of wolfberry by traditional methods due to the same variety, similar origins, and external characteristics. In the study, we have for the first time used a multi-task residual fully convolutional network (MRes-FCN) under Bayesian optimized architecture for imaging from visible-near-infrared (Vis-NIR, 400–1000 nm) and near-infrared (NIR-1700 nm) hyperspectral imaging (HSI) technology to establish a classification model for near geographic origin of Ningxia wolfberries (Zhongning, Guyuan, Tongxin, and Huinong). The denoising auto-encoder (DAE) was used to generate augmented data, then principal component analysis (PCA) was combined with gray level co-occurrence matrix (GLCM) to extract the texture features. Finally, three datasets (HSI, DAE, and texture) were added to the multi-task model. The reshaped data were up-sampled using transposed convolution. After data-sparse processing, the backbone network was imported to train the model. The results showed that the MRes-FCN model exhibited excellent performance, with the accuracies of the full spectrum and optimum characteristic spectrum of 95.54% and 96.43%, respectively. This study has demonstrated that the MRes-FCN model based on Bayesian optimization and DAE data augmentation strategy may be used to identify the near geographical origin of wolfberries.

## 1. Introduction

Ningxia wolfberries, the only medicinal wolfberry in China, are mainly distributed in Zhongning, Guyuan, Tongxin, and Huinong production areas. It is a genus of wolfberry in the Solanaceae family. Chinese wolfberry has a wide range of pharmacological effects, such as antioxidant, anti-fatigue, lowering blood lipids, enhancing the immune system, etc. The influence of environmental factors may result in significant differences in the quality and efficacy of wolfberries from different production areas in Ningxia [1]. Zhongning wolfberry, as the geo-herbalism representative of Ningxia wolfberries, has the best quality. The levels of medicinal components of Zhongning wolfberries, such as polysaccharides, are 10–70% higher than wolfberries of other origins. The contents of total soluble sugar and taurine are significantly higher than those in other regions. The levels of betaine, β-carotene, and flavonoids are significantly higher. Its price is also more than 30% higher than other regions of Ningxia [2]. The identification of the near geographical origin of wolfberries has become an important research area for the industry. Yin et al. [3] identified different varieties of wolfberries from relatively distant origins by electronic tongues. However, for the same variety of wolfberries, the similarity of latitude, climate, and the water source may further increase the difficulty of identification. The characteristics of targeted compounds in wolfberry are also affected by berry maturity and storage conditions. Traditional identification methods of wolfberry origin are mostly based on empirical identification, chemical analysis, traditional chromatography analysis, and complex mass spectrometry techniques [4]. They include isotope ratio mass spectrometry (IRMS), inductively coupled plasma mass spectrometry (ICP-MS), high-performance liquid chromatography (HPLC), gas chromatography (GC), and liquid chromatography-mass spectrometry (LC-MS/MS). These methods require skilled personnel to operate and are not only costly but also destructive to samples [5,6,7]. There are no significant differences in the levels of trace elements among different origins, so these techniques, such as ICP-MS, have limitations in the identification of the near geographical location of the fruit. Therefore, it is urgent to find an efficient, fast, and non-destructive method to identify the origin of wolfberries with the same variety.

Hyperspectral imaging (HSI) technology and machine vision have shown great potential in the field of adulteration identification. Xiao et al. [8] applied HSI to identify Radix Astragali from five different geographical origins. Studies by Fazari et al. [9] and Zhang et al. [10] found that image information from HSI enhanced model performance. Furthermore, in addition to the essential characteristics (endmember) and the corresponding fractions (abundance) in the spectrum, related optimization methods can also improve the performance of the model. The advances in chemometrics algorithms facilitate the development of HSI technology applications. Algorithm parameters optimization and algorithm improvements are important ways to improve the robustness of the model. Bayesian methods are becoming more popular because of their capacity to integrate prior information and efficient algorithms to estimate parameter distributions for complex models. Elegbede et al. [11] used a Bayesian network to optimize the sample size for food allergen monitoring. Li et al. [12] improved the classification accuracy of automatic electrocardiogram detection data with Bayesian optimization.

The application of deep learning combined with HSI is relatively backward in the food field. Most of the previous studies used 1D convolution kernel input to process HSI data [13]. In order to guarantee the size of the reception field, one-dimensional convolution often requires more kernels to learn and integrate features. However, the size of the effective receptive field (ERF) is proportional to the square root of the size and number of kernels. This means that it is more difficult to achieve large ERFs by stacking kernels [10]. Increasing the dimension of data was one of the focuses of this research. We converted the data into a two-dimensional image and then used up-sampling to increase the data dimension. The increase in data dimension also promoted the need for network depth. Simply deepening the network did not enhance performance. Despite the characteristics of the shallow network being replicated, the new layer as an identity map still leads to network degradation. A fully convolutional structure provides the possibility to continue to increase the depth of the model. The fully convolutional network (FCN) is a network structure in which all fully connected layers are transformed into convolutional layers. It avoids the problems of double computation and space waste caused by using neighborhoods. Sasank et al. [14] established a tumor growth model using FCN, and Wang et al. [15] achieved good results in dense forest image classification. The increase of convolutional layers may improve the network depth and model complexity. However, the classification effect is no longer significantly enhanced with increasing depth due to the saturation phenomenon. Therefore, a residual structure has been proposed to suppress gradient descent. Jiang et al. [16] used this structure for fluid flow predictions in large-scale geosystems. Zhao et al. [17] reported a plant disease classification model based on the fusion of inception and residual structure. The residual structure is usually used to improve the network depth, and this study pays more attention to its ability to capture overall information.

The multiple feature data collected by the two systems (Vis-NIR, NIR) are complex. Traditional data fusion strategies also lead to fuzzy features and increase the computational burden. This work first combined a residual network (Resnet) with a fully convolutional neural network (FCN) to fit these datasets. Its learning rate uses Bayesian optimization. Secondly, multi-task learning was introduced into the model, and a denoising auto-encoder (DAE)-based latent space disentanglement technique was used to enhance the learning of representation for novel data samples. Thirdly, principal component analysis (PCA) combined with gray level co-occurrence matrix (GLCM) was utilized to extract texture information from hyperspectral images of two different band ranges. We modified the architecture to implement a multi-feature classification complementary task that substantially improved the model performance by processing multiple data in parallel. The multi-task architecture used a shared backbone network. The generalization ability of the model was strengthened by eavesdropping and representation bias between multi-tasks [18,19,20]. Finally, all one-dimensional data were transformed into two-dimensional images. Transposed convolution was used to up-sample the image information. A classification model was built with MRes-FCN. In the past, the identification of the origin of wolfberries was mainly focused on the identification of different varieties of wolfberries with large differences in origin, mostly based on spectral information and conventional models. To our knowledge, the application of DAE to HSI data enhancement and the near geographic discrimination of wolfberries have not been reported yet. The study aimed to identify the near geographical origin of wolfberries by a combination of hyperspectral data and multi-task residual fully convolutional network.

## 2. Materials and Methods

### 2.1. Sample Preparation

**Figure 1:**
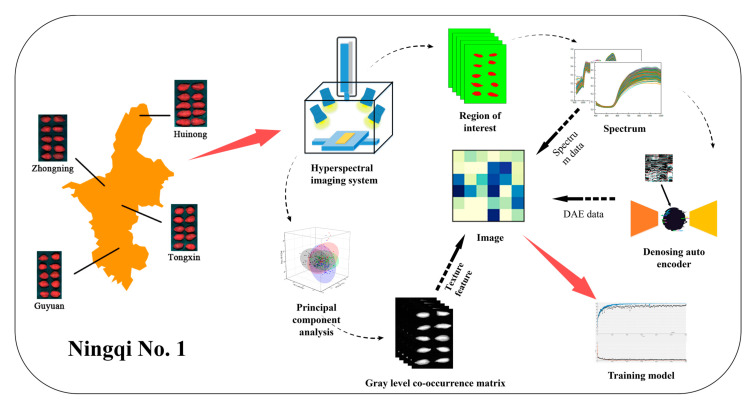
Flowchart of key steps in this study.

The wolfberries (variety of Ningqi No. 1, Figure 1) were from Huinong, Zhongning, Tongxin, and Guyuan production areas in Ningxia, China. The wolfberries of similar size, integrity, and with no obvious scars and deformation were chosen as samples. Every 1100 of the 4400 samples came from the same production area, and a total of 440 groups were generated by making every 10 wolfberries as 1 group.

### 2.2. HSI System and Image Calibration

The two sets of hyperspectral imaging systems (Zolix Instruments Co., Ltd., Beijing, China) mainly include a hyperspectral imager, a charge-coupled device (CCD) camera, four tungsten halogen lamps (35 W), an electronic displacement platform, and a computer. The position of the cameras and halogen lamps in the hyperspectral imaging system is shown in Figure 1. The wavelength ranges of the systems are 400–1000 nm (128 spectral bands) and 900–1700 nm (256 spectral bands), respectively. It is necessary to perform black and white calibration on the original hyperspectral image to avoid image noise caused by uneven light intensity and dark current in the process of hyperspectral acquisition. The correction equation was as follows.

(1) $$R=\frac{\;I-D}{W-D}\times 100\%$$

where R is the corrected spectral image, and I is the original spectral image of the samples. The dark reference image D was obtained by covering the camera lens cap (almost 0% reflectance); the white reference image W was obtained by a whiteboard made of PTFE material (>99% reflectance) [21].

### 2.3. Processing of Spectral Data

#### 2.3.1. Region of Interest and Spectral Data Extraction

The background of each sample was removed to obtain the average spectrum of 10 wolfberries in each hyperspectral image. The minimum (0.2) and maximum (1) thresholds were used to select the image. The region of interest (ROI) function of the ENVI 4.8 software was employed to extract the average spectrum of the sample from the background-free hyperspectral image. The same operation was performed on all samples and the average spectrum was used for analysis.

#### 2.3.2. Effective Wavelength Extraction

One of the important steps in processing spectral data is the extraction of effective wavelengths. In this study, interval variable iterative space shrinkage approach (iVISSA), competitive adaptive reweighted sampling (CARS), and uninformative variable elimination (UVE) were used to select the characteristic wavelengths. The iVISSA method is a search strategy combining local and global structures to obtain an effective wavelength interval with a set of optimized positions, combinations, widths, etc. The optimal wavelength was selected by CARS using the adaptive weighted sampling technique when the absolute value of the regression coefficients and the root mean square error of cross-validation (RMSECV) were minimized in the partial least squares (PLS) model for the spectral data. UVE obtained the regression coefficient matrix by PLSR cross-validation and used the mean of the regression coefficient vector with its corresponding standard deviation to filter the variable thresholds. 

### 2.4. Model Construction

#### 2.4.1. Multi-Task Residual Fully Convolutional Network

**Figure 2:**
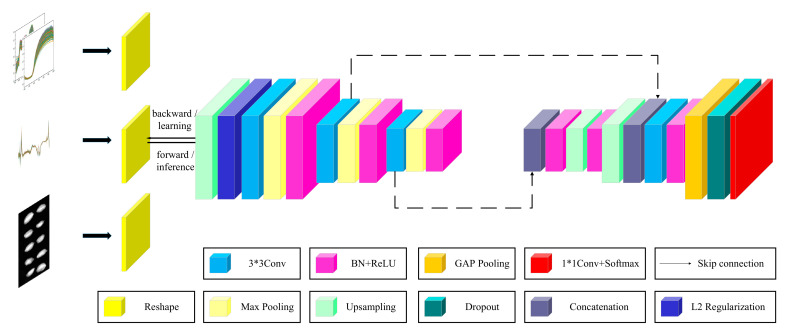
MRes-FCN+ based multi-task network architecture.

Our architecture (Figure 2) can be viewed as a type of multi-task learning architecture. In this study, all fully connected layers in the convolutional neural network (CNN) were replaced by convolutional layers to form FCN. The convolutional layer was expressed by the following equation:

(2) $$y^{j}=f\left(b^{j}+\sum_{i}k^{\mathrm{ij}}{*x}^{i}\right)$$

where x^i^ and y^j^ are the i-th input graph and the j-th output graph, respectively. k^ij^ is the convolution kernel between the mapping i and j, ∗ represents the convolution, and bj is the deviation parameter of the j-th mapping. MRes-FCN consists of a single shared encoder that learns general spectral features and two task-specific decoders that learn spectral and textural features concerning each specific task. Cross entropy has some mathematical properties that make it a good choice for handling this classification problem [9].

**Figure 3:**
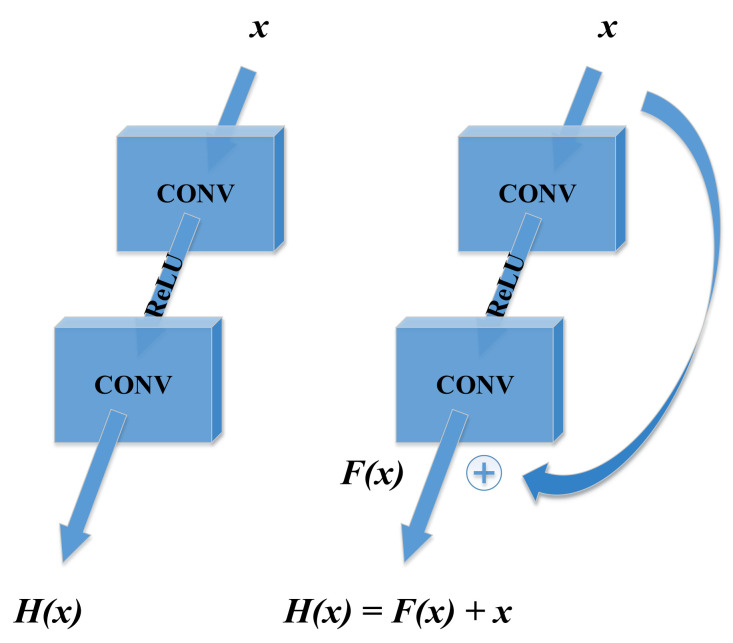
A regular block (**left**) and a residual block (**right**).

Residual blocks help solve the problems of gradient disappearance and gradient explosion [16]. The difference between them and the regular block is shown in Figure 3. The proposed network consisted of a basic core network and an attention module, which could capture global information. 

A combination of HSI and deep learning in the past was restricted by datasets and the effective features of the one-dimensional convolution extraction model. Thus, in this study, the fully connected dimension added method was used to obtain two-dimensional images. The size of feature maps was amplified by up-sampling. The end of the network used a 1 ∗ 1 convolutional layer and softmax to output the result. More details of the other layers are described in Section 2.4.3.

#### 2.4.2. Algorithm Optimization

In the deep learning model, gradient descent optimizes the weight coefficients and deviations to keep the loss function as small as possible [21]. The learning rate is the most important hyperparameter [8,9]. It directly affects the gradient convergence rate and iteration times. A few methods are created and studied to find the proper combination of the learning rate. These methods include whale algorithms and random search. However, these methods have disadvantages, such as low accuracy, slow convergence, and a tendency to fall into local optimality. Compared with these methods, Bayesian Optimization is better for learning rate searching [11]. Bayesian methods take into account available information by using prior distributions for model parameters, rather than a point estimate, so uncertainty is taken into account in the results [12].

**Figure 4:**
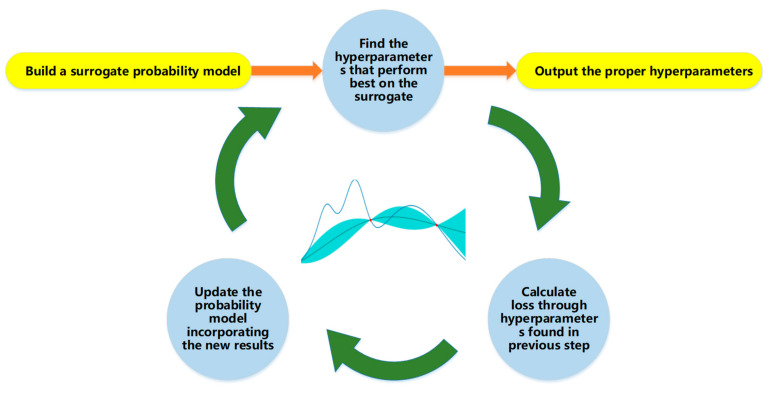
Process of Bayesian optimization.

The Bayesian framework was originally applied to the optimization of parameters of a neural network model, with good results [22]. The Bayesian evidence framework was used to obtain the optimal parameter values by maximizing parameter distribution [23]. The whole process of Bayesian Optimization is shown in Figure 4.

#### 2.4.3. Avoiding Overfitting

The model generalization performance was reduced by excessive HSI features, overtraining, and the presence of noise. To solve such problems, we used the following methods: L2 regularization, sparse processing, batch normalization (BN), and dropout. 

According to the principle of Occam’s razor, appropriately reducing the model complexity has a significant effect on reducing overfitting. Regularization is a technique to prevent this problem by introducing additional information [24,25]. As opposed to L1-norm regularization which is often used to feature selection, it is easier to optimize the objective function with the L2-norm regularization [26]. A key reason for choosing sparse processing is that it can automatically select features and help reduce learning difficulties [27]. The BN layer can standardize data training through a transformation and reconstruction algorithm. The output data of each layer is maintained to a fixed mean and standard deviation, so it will prevent gradient dispersion and gradient explosion. Dropout can significantly reduce overfitting by ignoring a certain number of feature detectors in each training batch. This network added it as a penalty term to the initial fully connected layer. The sparse process was completed at the transposed convolutional layer. BN layers were added after the convolutional layers of all models, and a dropout with a preset probability of 0.4 was added between the final merged layer and the fully connected layer. In addition, the sample sets of data were divided into two datasets in a ratio of 3:1 according to the sample set partitioning based on joint x-y distance (SPXY), and all models were constructed for these datasets.

### 2.5. Multi-Task Data

#### 2.5.1. Denoising Auto-Encoder (DAE)

**Figure 5:**
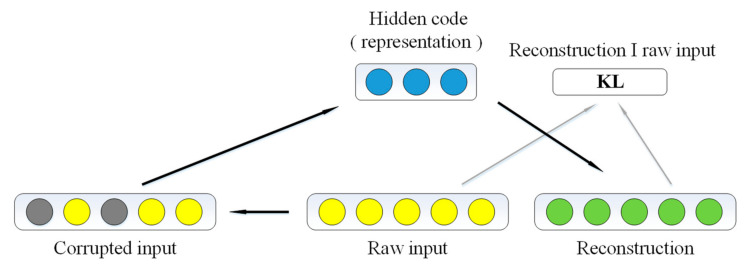
The architecture of denoising auto-encoder.

Appropriate datasets are necessary for the multi-task model. Hidden layer features were obtained by using DAE. It is a robust variation of AE, in which a stochastically corrupted version of the input is used to feed the AE (Gaussian additive noise with zero means), while the uncorrupted input is still used as the target for the optimization of the parameters [28,29]. The general idea is depicted in Figure 5.

#### 2.5.2. Spectrum Pre-Processing

In this work, the following pre-processing methods were used: Savitzky-Golay (SG, 2nd order polynomial, 25 points), Normalize, multiplicative scatter correction (MSC), orthogonal signal correction (OSC), and the first derivatives and the second derivatives (1st Der and 2nd Der). These methods generated data with more characteristics. These data were used for comparison with DAE data. It was the main basis for judging the contribution of the DAE to this work.

#### 2.5.3. Extraction of Textural Data

**Figure 6:**
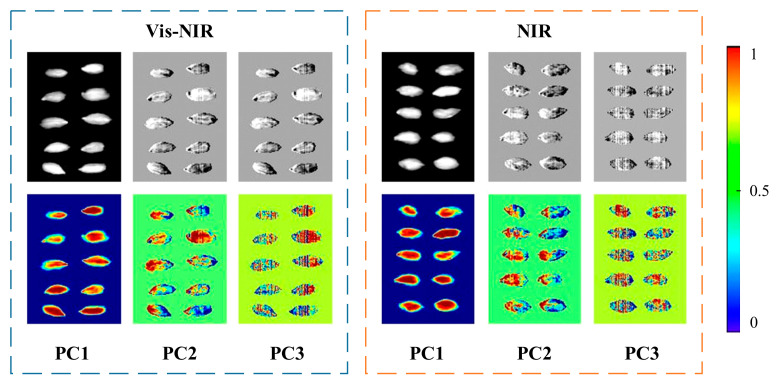
PCA of pseudo-color visual images.

Texture information is an important indicator to reflect the external characteristics of the samples [30]. Grayscale co-occurrence matrix (GLCM) is a texture feature (TF) extraction method based on the spatial distribution relationship between pixels [31]. In this work, principal component analysis (PCA) was used to select feature images to extract TF. Figure 6 shows the principal component images of the first three principal components in the PCA of Vis-NIR and NIR and their reflectance pseudo-color visualization images. PC1 covers most of the information in the sample, and its cumulative spectral variances were 82.21% and 85.88%, respectively (Figure S1). We extracted 4 parameters (angle second moment, entropy, homogeneity, and correlation) in PC1 images. Generally, the increased feature dimension after a certain critical point may lead to the Hughes effect in practical applications [32]. Therefore, we used the variance and standard deviation of the 4 feature parameters to represent the image features.

### 2.6. Model Evaluation

Accuracy is used as the main evaluation index in most studies because of its interpretability. Generally speaking, the average accuracy of the model should be close to 100%, and the standard deviation should be as small as possible. However, it is not rigorous to determine the final performance of the model only by the accuracy [9]. Therefore, in addition to accuracy, precision (PRC), recall (RC), and specificity (SPC) scores were calculated to comprehensively evaluate the practicability of the model. Equations (3)–(6) are the calculation methods of several evaluation indicators.

(3) $$\mathrm{Accuracy}\left(\%\right)=\frac{\mathrm{TP}+\mathrm{TN}}{\mathrm{TP}+\mathrm{FP}+\mathrm{FN}+\mathrm{TN}}\times 100\%$$

(4) $$\mathrm{Precision}\left(\%\right)=\frac{\mathrm{TP}}{\mathrm{TP}+\mathrm{FP}}\times 100\%$$

(5) $$\mathrm{Recall}\left(\%\right)=\frac{\mathrm{TP}}{\mathrm{TP}+\mathrm{FN}}\times 100\%$$

(6) $$\mathrm{Specificity}\left(\%\right)=\frac{\mathrm{TN}}{\mathrm{TN}+\mathrm{FP}}\times 100\%$$

where TP is a true positive, representing a positive sample predicted by the model as a positive class; TN is a true negative, indicating a negative sample predicted by the model as a negative class; FP is a false positive indicating a negative sample predicted by the model as a positive class and FN is a false negative means a positive sample predicted by the model as a negative class. The data analysis software was MATLAB 2020b, ENVI 4.8, The Unscrambler X10.4, Origin 2020, and Python3.8. 

## 3. Results and Discussion

### 3.1. Overview of Spectral Profiles

**Figure 7:**
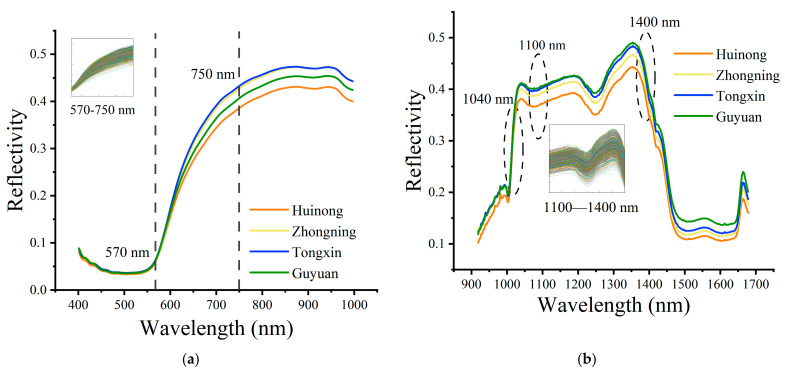
Vis-NIR (**a**) and NIR (**b**) average spectral curve of different wolfberry production areas in Ningxia.

As shown in Figure 7, the spectral reflectance curves of wolfberries from different regions were roughly the same. The spectral reflectance of wolfberries was relatively low in the range of 400–570 nm, and the spectral curves of each group of samples tended to overlap (Figure 7a). Due to the color of the surface of wolfberries, the reflectance of visible light wavelength in the range of orange to red (570–750 nm) gradually increased, and slight differences in spectral curves gradually appeared [7]. The NIR (Figure 7b) can reflect more molecular structure and composition information of the substance. The peaks and valleys of the curve mainly correspond to the frequency doubling, and the combination of the transition from the ground state to the high vibrational energy level is caused by the molecular vibration, mainly including hydrogen-containing groups. After removing the bands with noise at the front and back, it was observed that the curve begins to diverge after the first absorption peak at 1040 nm, which is due to the second vibration of the N-H bond in the protein or amino acid. There were significant differences in the spectral curves from 1100 nm to 1400 nm, which are close to the double-frequency absorption band of the C-H bond [6]. The absorption peak is due to the secondary stretching vibration of the C-H bond in the protein, starch, or lipid. After 1400 nm is the sensitive area for water absorption. As the dried wolfberries were used in the study, the reflectivity of the sample was not obvious.

Although there were differences between the spectral curves at specific wavelengths for samples from different origins, there were overlaps between the curves, and the differences in different wavelength bands were small, so it was difficult to classify by observation alone. Therefore, it is necessary to further analyze the data to determine the origin of wolfberries.

### 3.2. Optimization of Model Parameters

**Figure 8:**
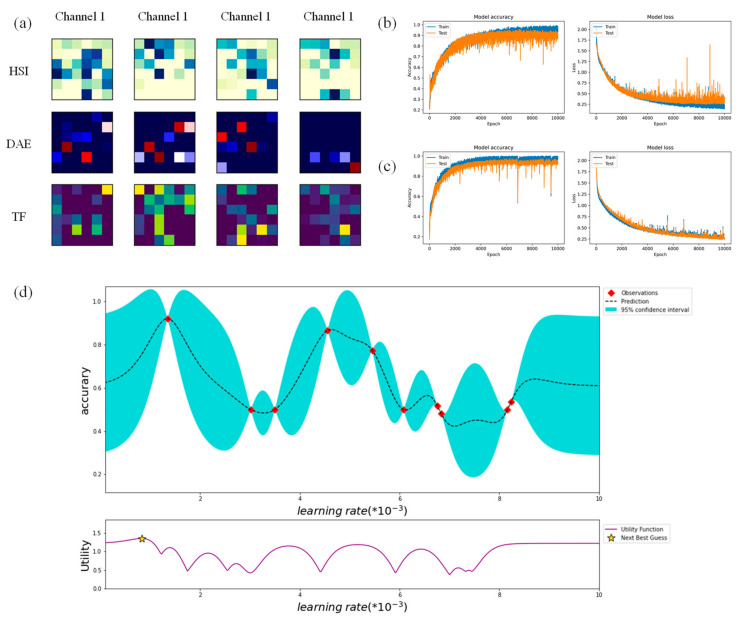
(**a**) Image from visualization after reshaping three sets of data. The three rows in the figure represent the datasets for the three tasks, and the columns represent the channels; (**b**,**c**) are model effects using 1D convolution and up-sampled images, including accuracy and loss function. (**d**) Bayesian optimization curve. Where cyan is the function range of the Gaussian process, red is the collection point through which all the functions it produces will pass. The dashed line is the mean of these functions. The purple curve below the image predicts the next point in the optimization process.

The up-sampling was compared with conventional sampling in the study. A two-dimensional visualization image after the input data was reshaped (Figure 8a). These image textures represent differences in the learning characteristics of different data and channels. The model using 1D convolution sampling (Figure 8b) had a slower convergence speed, with an accuracy of 0.8 at epoch = 2000. The model using transposed convolution (Figure 8c) was faster and more stable. Its precision curve converged to 0.86 when its epoch reached 2000. This may be because the 1 ∗ 1 convolution kernel cannot represent the features of sparse data, resulting in reduced learning efficiency. The results showed that reshaping data and using up-sampling were more suitable for this study. 

The mean value of the Gaussian function was stable during the exploration period (Figure 8d). According to the purple curve in the figure, the collection point of the next round may be lower than the current one. After 10,000 rounds of Bayesian exploration, the best result was obtained at a learning rate of 0.0012, which appeared at the first collection point on the dotted line. 

### 3.3. Effective Wavelengths

**Figure 9:**
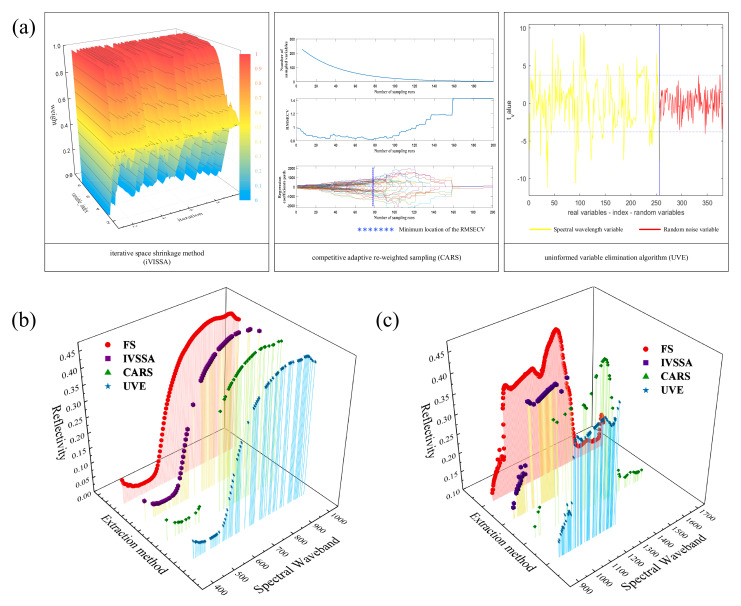
(**a**) Methods of characteristic wavelengths extraction; (**b**,**c**) are the results of Vis-NIR and NIR characteristic wavelength extraction, respectively. Red line represents the full spectrum (FS).

Two different spectral ranges (400–1000 nm and 900–1700 nm) were used to collect data in the study. In order to reduce information redundancy, we adopted three methods to screen the characteristic wavelengths (Figure 9a). In the VIR-NIR hyperspectral system (Figure 9b), iVISSA and UVE reduced it to 72 and 74 wavelengths, respectively. The extracted wavelengths were mainly distributed in the parts with obvious curvature. The 42 wavelengths extracted by the CARS method were more concentrated in the red and near-infrared scopes. The curves for the different samples started to diverge around here. The results indicated that the difference in reflectance intensity was more of a concern to CARS. iVISSA selected 123 useful wavelengths in NIR (Figure 9c). These accounted for 48% of the total wavelength variation. Most of them were in the higher reflectivity parts. The CARS method screened 42 wavelengths. Similar to Vis-NIR, they were mostly in the regions with large spectral differences between samples. The 64 wavelengths extracted by UVE were the densest at the wave peaks and valleys. This result also confirmed that UVE was more inclined to extract parts with obvious curvature.

### 3.4. Modeling Results

#### 3.4.1. Comparison of Multi-Task Datasets

Table 1 shows the effect of adding DAE and other preprocessed data to the multi-task model. These results demonstrated the effectiveness of DAE data for multi-task models. Its overall effect was significantly higher than other data obtained by preprocessing. The results from the training sets and test sets using DAE data were improved by 6.12% and 6.16%, respectively. In addition, the test sets of 1st Der were also improved by 1.77%. This is due to its feature amplification effect, but it also causes instability in training. SG and OSC were improved by 8.87% and 9.48%, respectively in the training set, but they had the obvious overfitting phenomenon. This phenomenon proved that transition noise removal reduced the generalization ability of the model. Noise prevented the network from remembering training samples and made it more robust.

#### 3.4.2. Comparison of Modeling Results

**Figure 10:**
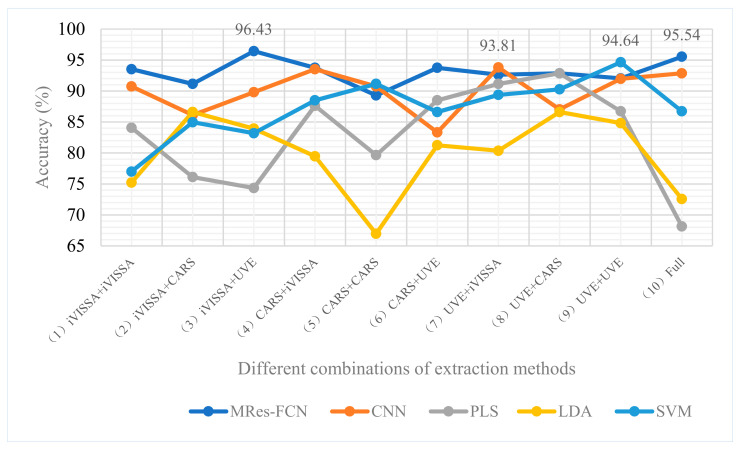
The classification results after extracting the characteristic wavelengths of the two spectral ranges, respectively. Different combined extraction methods were marked with 1 to 10.

In this study, MRes-FCN was used to compare with Convolutional Neural Network (CNN), Partial Least Squares (PLS), Linear Discriminant Analysis (LDA), and Support Vector Machine (SVM) on the same characteristic spectral dataset. As shown in Figure 10 the best performing MRes-FCN (Group 3) model yielded an accuracy of 96.43%, while its full-spectrum (Group 10) data achieved 95.54%, followed by CNN (Group 7) and SVM (Group 9). In addition, the results indicated that traditional models were more suitable for processing extracted data. Extracting characteristic wavelengths reduced the complexity of the data, which limited some learning advantages of black-box models. Compared to deep learning models, traditional models improved the computational efficiency and the running speed, although most of the classification accuracies were reduced. Even so, the model was sufficient to classify Huinong, Zhongning, Tongxin, and Guyuan wolfberries. Therefore, to further determine the application potential of the model, a more detailed analysis of the results is required.

### 3.5. Model Evaluation

FIt is not rigorous to rely solely on the accuracy to evaluate the effect of a relatively close result in this study. Therefore, we calculated evaluation metrics for each of the four modeling results in the study (Figure 10; Table 2). The Guyuan sample had lower PRC in all results, with the lowest being 78.57%. This may be related to the geographical location of Guyuan and Tongxin. The closeness of the soil and water environment of the two origins may lead to a high similarity in characteristics. Part of Tongxin wolfberry was classified into Guyuan by the model to cause misjudgment, which was confirmed by its lower RC. The reason why some of the real samples were not correctly predicted each time was that Tongxin’s RC did not reach 100% in all results. 

Generally, high accuracy can bring production efficiency and economic benefits for the origin traceability. However, the SPC of Zhongning wolfberries classification is more important than accuracy, because the SPC is not high, meaning that other production areas may be classified as Zhongning wolfberries sold at a high price, resulting in the collapse of business reputation and brand value. As shown in Table 2, all models showed good application potential in identifying Zhongning wolfberries. MRes-FCN (Group 3), MRes-FCN (Group 10), and CNN (Group 7) all achieved 100% SPC. Among them, the PRC and RC of MRes FCN (Group 3) also reached 100% and 96.55%, respectively. Kappa coefficients of four sets of results, to measure their classification effects, were calculated in the supplementary data of Table S1. These results are similar to other studies on the original classification of wolfberries [2,3,4,5,6,7], but the previous studies on the classification of wolfberries were of different varieties. For example, LS-SVM was used by Li et al. [2] to calibrate the discriminative model of superior quality and inferior quality black wolfberries in Luo Mu Hong, Qinghai-Tibet Plateau, and Xinjiang; Shen et al. [4] used near-infrared spectroscopy and chemometrics to determine the geographic origin of wolfberries in the North China Plain, Loess Plateau, Northeast China Plain, and the Northwest Basin; Mu et al. [6] classified HSI images of wolfberries from four origins in Ningxia, Qinghai, Xinjiang, and Gansu. These origins have relatively far distances, large differences in water and soil environment and growth conditions, and so can be identified easily. In this study, we achieved good results in the more difficult near-geographic classification, demonstrating its application value in the market. Although the transposed convolution has learned effectively on the original data, feature extraction and data enhancement strategy also obtained outstanding results, which indicates that these are feasible strategies that could effectively distinguish the origin of the same variety of wolfberries with smaller feature gaps. 

## 4. Conclusions

In this study, using HSI combined with deep learning we compared a variety of data fusion strategies to identify several major production areas of wolfberries in Ningxia. It was found that the combination of data reshaping methods and transposed convolutional input data can significantly improve the stability of the model and reduce overfitting. At the same time, the training cost of the model was reduced by the addition of Bayesian optimization. The enhanced data generated using DAE provided a new option for training multi-task models. The results showed that the datasets combining iVISSA and UVE feature extraction were the best modeling effect on MRes-FCN. Its ACC (all origins) and SPC (Zhongning) reached 96.43% and 100%, respectively. This indicates that MRes-FCN is more advantageous in the complex processing of high-dimensional data compared to traditional modeling methods. Compared with earlier data processing methods, the MRes-FCN structure had higher accuracy and sensitivity with complex data. Therefore, the model training method and data fusion strategy adopted in this study are effective methods to determine the near geographic origin of wolfberries in Ningxia.

The multi-task structure used in this work improved the efficiency of traditional data fusion. The data reshaping method may provide new possibilities for future HSI combined with deep learning detection research. However, MRes-FCN has high hardware requirements. The applicability of the method can be further improved if the trained network can be moved to an embedded device, such as a Raspberry Pi without loss of accuracy.

## Figures and Tables

**Table 1:** Comparison of various data addition models, the first column (Basic) is the result of not using these methods to augment the data.

	Basic	SG	OSC	Normalize	MSC	1st	2nd	DAE
Train (%)	89.60	98.47	99.08	89.30	87.16	93.88	82.26	95.72
Test (%)	89.38	91.15	92.59	93.81	90.74	91.15	81.25	95.54

**Table 2:** Model evaluation indexes of different producing areas.

	MRes-FCN (Group 3)				MRes-FCN (Group 10)				CNN (Group 7)				SVM (Group 9)			
	ACC (%)	PRC (%)	RC (%)	SPC (%)	ACC (%)	PRC (%)	RC (%)	SPC (%)	ACC (%)	PRC (%)	RC (%)	SPC (%)	ACC (%)	PRC (%)	RC (%)	SPC (%)
Huinong	96.43	100	96.55	100	95.54	100	93.33	100	93.81	100	93.33	100	94.64	100	90.32	100
Zhongning		100	96.55	100		100	93.33	100		100	93.33	100		96.43	93.10	98.75
Tongxin		100	96.55	100		100	93.33	100		96.43	90	98.73		100	96.55	100
Guyuan		85.71	100	95.45		82.14	100	94.38		78.57	100	93.26		82.14	100	94.32

## Data Availability

Data is contained within the article or supplementary material.

## Supplementary Materials

The following supporting information can be downloaded at: https://www.mdpi.com/article/10.3390/foods11131936/s1, Figure S1: Scatter plot of 3D principal component scores for Vis-NIR (a) and NIR (b); Table S1: Results of Kappa coefficient.
