# Supplementary material for: Identification of Near Geographical Origin of Wolfberries by a Combination of Hyperspectral Imaging and Multi-Task Residual Fully Convolutional Network
Source: Foods. 2022 Jun 29;11(13):1936. doi: 10.3390/foods11131936 (PMC9265825; doi:10.3390/foods11131936)
Supplement: Supplementary file 1 [file foods-11-01936-s001.zip › foods-1751956-supplementary.pdf]

Supplementary

Supporting Information

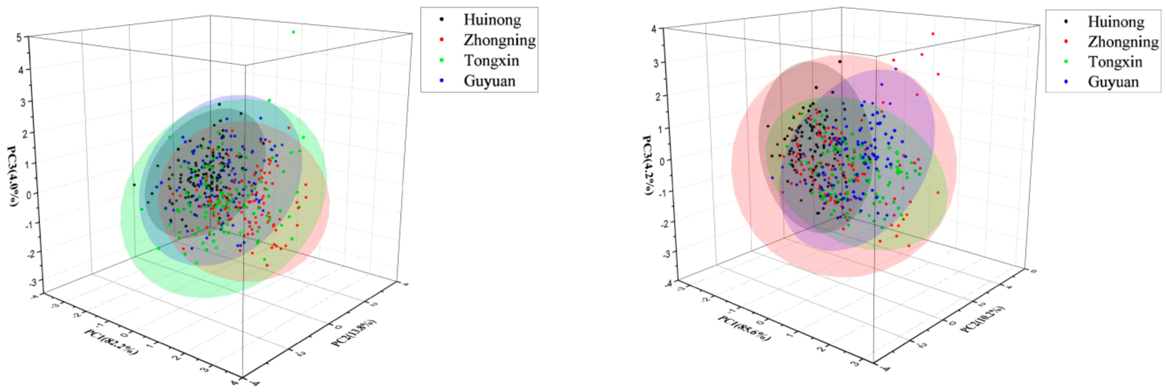

(**a**) (**b**)

Figure S1. Scatter plot of 3D principal component scores for Vis-NIR (a) and NIR (b).

Table S1. Results of Kappa coefficient.

	MRes-FCN (Group 3)	MRes-FCN (Group 10)	CNN (Group 7)	SVM (Group 9)
ACC (%)	96.43	95.54	93.81	94.64
Kappa	0.9524	0.9405	0.9174	0.9285
